# Improvement of Pit-and-Fissure Sealant Bonding to Enamel with Subpressure Treatment

**DOI:** 10.1155/2019/5070383

**Published:** 2019-03-28

**Authors:** Chang Zhang, Yongmei Li, Zutai Zhang, Yueming Tian, Ning Ding, Yongping Ma

**Affiliations:** ^1^Capital Medical University, School of Stomatology, Beijing 100050, China; ^2^Beijing Institute of Dental Research, Capital Medical University, School of Stomatology, Beijing 100050, China; ^3^Department of Stomatology, The No.2 Hospital of Baoding, Baoding, Hebei 071000, China

## Abstract

This research evaluated the effects of subpressure on the shear bond strength (SBS) of 80 specimens with flat enamel surfaces and on AgNO_3_ microleakage of 40 specimens with flat enamel surfaces and 40 specimens with 1 mm deep cavities before and after thermocycling. The enamel of 168 specimens was grounded to a flat surface. Two types of sealants (E and H) were selected. Sealants were applied to enamel surface (88 specimens, group F) either subjected or not to subpressure. The bonding interfaces were observed using scanning electron microscopy (SEM) and the SBS was examined using a universal testing machine before and after thermocycling. The failure mode was also analyzed. For the microleakage test, 80 specimens were grouped as group A (original enamel flat surface) and group B (a round cavity of 1 mm in depth) (40 per group). Sealants were applied to the teeth either subjected or not to subpressure. The specimens were submitted to a microleakage protocol with AgNO_3_ and analyzed before and after thermocycling. Statistical analysis was performed for the data. The results showed that subpressure eliminated voids on the interface between the enamel and sealants and significantly enhanced specimens' SBS. Although thermocycling reduced SBS significantly, specimens under subpressure after thermocycling still showed higher SBS than specimens under nonsubpressure before thermocycling. The subpressure groups showed a lower microleakage level compared to nonsubpressure groups, though thermocycling caused deeper silver infiltration. In addition, different sealants showed no significant effect on the SBS and microleakage performance. Overall, subpressure application improves sealant bonding and retention rate and has potential to prevent secondary caries.

## 1. Introduction

As the National Health and Nutrition Examination Study (NHANES) 2011-2012 data indicate, pit-and-fissure caries comprise about 90% of the total caries in permanent posterior teeth and 44% of total caries in primary teeth in children and adolescents [1]. The plaque retentive nature of pits and fissures makes them tough to clean, which causes smooth surfaces not to be more susceptible to caries than pits and fissures [2]. In the past, a few efforts had been made in order to shield pits and fissures against caries. Methods such as topical fluoride therapy, community water fluoridation, dietary sugar control, and plaque control have been in general considered as the primary causes for the total decrease in caries prevalence, which in turn greatly reduces the occurrence of smooth surface carious lesions [3, 4]. Other approaches such as enamel fissure eradication or alleged fissurotomy could transform deep pits and fissures into cleansable ones. This approach, however, involves the widening of the fissures [5]. In the clinic, dentists frequently encounter the dilemma that an invasive ‘‘biopsy” is carried out in order to evaluate the extent of caries and restore the teeth [6]. Therefore, young patients with caries activity are clinically advised to adopt preventive pit-and-fissure sealing. And pit-and-fissure sealing has been considered as the standard procedure to prevent caries [7]. Pit-and-fissure sealing in preference to no sealant or fluoride varnish is recommended by the recent evidence-based guidelines of the American Dental Association (ADA) and the American Academy of Pediatric Dentistry (AAPD) [8]. In order to keep bacteria away from their source of nutrients, sealants can bond the enamel micromechanically to provide a physical barrier [9, 10].

However, it has remained a tendency of secondary caries forming at the margins of sealants despite proven clinical benefits of pit-and-fissure sealing [11]. And lower retention and marginal staining are still worthy of consideration [12]. The reason why sealants can decrease the incidence of caries is that sealants have high penetration ability and can continuingly resist microleakage [13]. Studies have found that the acid-etched enamel surface exposed after the sealant shedding is more susceptible to bacterial damage than the normal nonetched enamel surface [14, 15]. Valid protection will carry on so long as the sealant materials remain bonded to the enamel. As a result, the retention of sealants and resistance of microleakage become the true determinant [16]. Lately, the report of Zhuge has shown that the depth of resin tags inside the dentinal tube can be improved by the subpressure technique [17]. Tian also claims that the penetration of pit-and-fissure sealants can be increased by subpressure treatment and resistance demineralization of pit-and-fissure sealants can be increased as well [18]. This paper aims to evaluate the influence of subpressure on the bonding of sealants and resistance of microleakage before and after thermocycling. The null hypothesis of this study is that subpressure can enhance the bonding of sealants-enamel and reduce the microleakage.

## 2. Materials and Methods

### 2.1. Specimen Preparation

After the patients' informed consent and the approval of the local ethics committee were obtained, 168 human molars were assembled. These molars were stored in a normal saline solution before use [19]. After the roots of the teeth were cut off, the crowns were embedded in acrylic resin and the buccal enamel surfaces were exposed. In order to obtain flat enamel surfaces with diameters of at least 3 mm, the buccal enamel surfaces were grounded with water-cooled silicon carbide sandpaper (400-, 800-, and 1200-grade paper). 88 specimens with the original flat enamel surface were assigned to group F and were used for interface observation and the shear bond strength (SBS) test; 40 specimens with the original flat enamel surface and 40 specimens with round cavities of 2 mm in diameter and 1 mm in depth prepared with a dental handpiece were assigned to group A and group B, respectively. And the specimens of group A and group B were used for microleakage evaluation. Two types of sealants were evaluated in this study, and they were sealant 3M ESPE Concise (3M ESPE Dental Products, McGaw, USA; Code E) and sealant Helioseal F (Ivoclar Vivadent AG, USA; Code H). Therefore, these specimens of groups F, A, and B were further subgrouped as groups FE, FH, AE, AH, BE, and BH with 44, 44, 20, 20, 20, and 20 specimens, respectively.

### 2.2. Interface Observation and SBS Test

**Figure 1:**
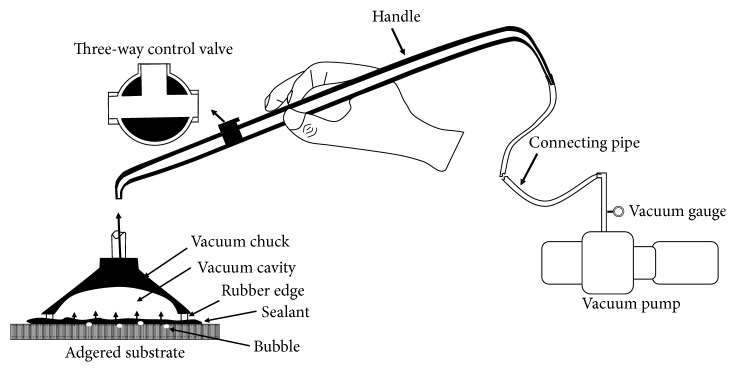
The illustration of the experimental subpressure apparatus.

A piece of 50 *μ*m thick scotch tape with a circular hole of 3 mm in diameter was placed on the flat enamel surface of specimens in groups FE and FH (44 per group) to delimit their bonding area. And the sealant was applied to the enamel surface of the hole according to the manufacturer's introduction. These specimens were subjected to either subpressure (code S) or nonsubpressure (code N) treatment and further subgrouped as groups FE_N_, FE_S_, FH_N_, and FH_S_ with 22 specimens per group. After the sealants were applied to the enamel surface, specimens of the subpressure groups (groups FE_S_ and FH_S_) were placed into an experimental subpressure apparatus, while sealants were applied to specimens of the nonsubpressure groups (groups FE_N_ and FH_N_) according to the manufacturer's introduction. The experimental subpressure apparatus which was used to provide the subpressure condition in this experiment was made up of four parts: a vacuum chuck, a hollow handle, a vacuum pump, and a governor valve. In order to provide a seal between the vacuum chuck and enamel, the vacuum chuck had a rubber edge. The dimensions of the vacuum chuck must be adequate to satisfy the need for sealing. A three-way valve was contained on the hollow handle, which could switch the passageway to the pump or outside. A vacuum gauge and a vacuum relief valve were included on the governor valve. The relative vacuum degree could be adjusted by the vacuum relief valve, and the subpressure degree could be obtained by the vacuum gauge. The vacuum pump was used to provide the vacuum. A connecting pipe was used to connect the governor valve and the hollow handle. Under the subpressure condition, the edge of the vacuum chuck and the enamel tightly contacted each other. The control valve was used to switch on and off the vacuum pump, and the relative subpressure degree in the vacuum chuck was controlled by the vacuum relief valve. When the control valve was switched to the pump side, the pump was turned on and subpressure condition was created. And when the control valve was switched to the outside, the subpressure condition was removed. The experimental subpressure apparatus was illustrated in Figure 1.

For specimens in the subpressure groups (FE_S_ and FH_S_), the vacuum chuck was tightly placed over the plane of the hole after the sealant was applied. The subpressure switch was turned on until the inside pressure of the chuck was dropped to -0.1 MPa. After holding the subpressure condition for 15 s, the vacuum pump was turned off and the vacuum relief valve was used to remove subpressure and return to the nonsubpressure condition.

The precast composite resin (Valux™ Plus, 3M ESPE, USA) columns with diameter of 3 mm and thickness of 4 mm in a steel mold was put on the sealant surface to cover the hole of scotch tape. Then, 20 N of force was loaded on the resin column, with a holding time of 1 min. And conventional curing was conducted for 20 s using a light curing unit (Elipar™ 2500, 3M ESPE, USA). After finishing the sealant curing, the four groups FE_N_, FE_S_, FH_N_, and FH_S_ (88 specimens) either subjected or not to thermocycling (instant and thermocycling, codes I and T) were further subgrouped as FE_NI_, FE_SI_, FH_NI_, FH_SI_, FE_NT_, FE_ST_, FH_NT_, and FH_ST_. Specimens in groups FE_NI_, FE_SI_, FH_NI_, and FH_SI_ were instantly used for subsequent testing. Specimens in groups FE_NT_, FE_ST_, FH_NT_, and FH_ST_ were subjected to thermocycling. The thermocycling was under the condition of 5000 cycles of increasing temperature from 5°C to 55°C at a dwell time of 30 seconds per temperature and a transfer time of 5 seconds between baths (TC-501F, WELL, Suzhou, China).

One specimen from each group was selected for the bonding interface observation. They were sectioned perpendicularly to the bonding surface by a water-cooled diamond disk (Isomet 4000 Linear Precision Saw, Buehler, USA). And the bonding interfaces were observed by a scanning electron microscope (SEM) (Phenom-World Co., Ltd., Netherlands) at 5,000x.

The rest of the 10 specimens from each group were estimated by SBS with a universal testing machine (AG-X Plus, Shimadzu Co., Ltd., Shimadzu, Japan). The load was applied at 1 mm/min until failure occurred. The shear bond strength was calculated according to the formula

(1) $$\begin{array}{c} \sigma =\frac{F}{S} \end{array}$$

where *σ* is shear bond strength (MPa), F is maximum load shear force (N), and S is bonded area (mm^2^).

After the SBS test, the deboned enamel surfaces were inspected under a SEM at magnification of 3000x at accelerating voltage of 15 kV and beam current of 110 *μ*A. The fracture modes were classified into three types [20]. The first one is cohesive fracture, which is defined as internal fractures of the enamel or of the resin/sealant. The second one is adhesive failure, which is defined as the complete delamination of sealant from the enamel and the surface between the enamel and sealant, showing very little sealant on the enamel side and lack of enamel on the resin side. The third one is the combination of cohesive fracture and adhesive failure, or the mixed failure, in which the enamel of the tooth has a large amount of sealant and resin, and the resin side retains a large amount of the enamel.

### 2.3. Microleakage Evaluation

**Figure 2:**
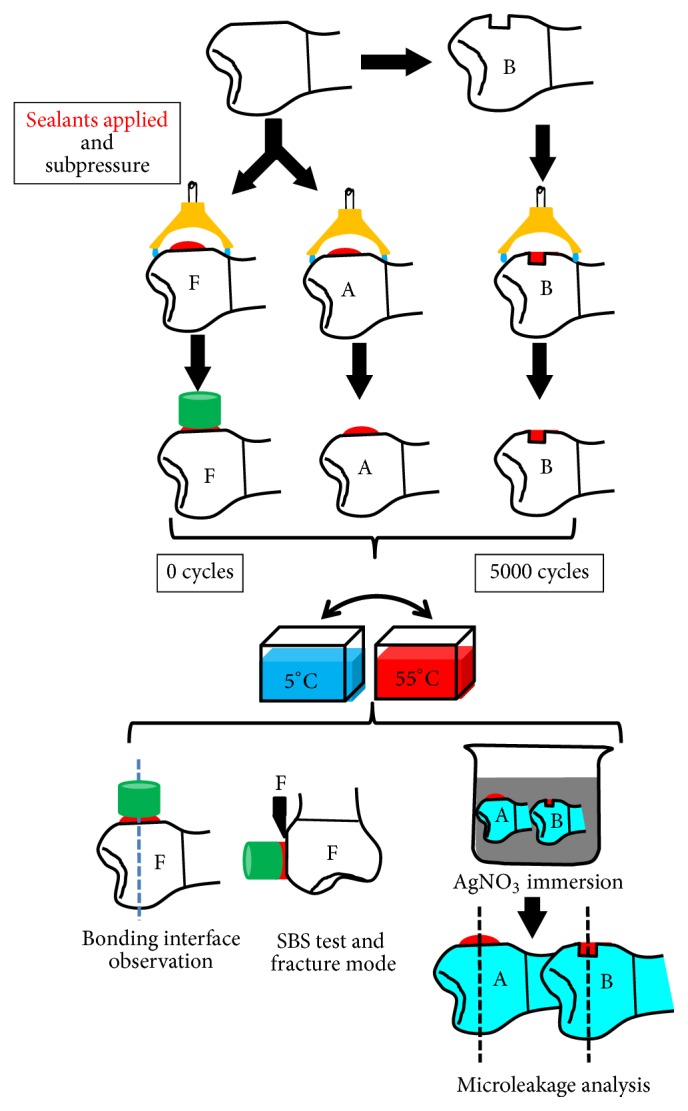
Schematic diagram of the study (nonsubpressure groups as control).

Specimens in groups AE, AH, BE, and BH were used for the microleakage test (20 per group). In brief, a drop of sealant about 2 mm in diameter was applied to the enamel surface of specimens in groups AE and AH. The sealants were applied into the cavity of specimens in groups BE and BH, and meanwhile the sealant surface was flush with the cavity edge. Among these samples, half in each group were subjected to subpressure after the sealants were applied. And they were named as groups AE_S_, AH_S_, BE_S_, and BH_S_ (5 per group). The subpressure condition was as described above. The sealants were applied under the nonsubpressure condition in the other half of specimens in each group, and they were assigned into groups AE_N_, AH_N_, BE_N_, and BH_N_ (5 per group). After finishing sealant curing, half of the specimens in each group were named as groups AE_NI_, AH_NI_, BE_SI_, and BH_SI_ and instantly used for subsequent testing. The rest of the specimens in each group were assigned into groups AE_NT_, AH_NT_, BE_ST_, and BH_ST_ and subjected to thermocycling. The thermocycling condition was as has been noted above. The surface of each specimen was covered by two layers of nail varnish, 1 mm far from the bonding interface. All specimens were immersed in 50 wt% AgNO_3_, holding for 24 hours at 37°C. After water rinsing for 2 min, the specimens were immersed in a photo developing solution for 8 hours. After water rinsing, these specimens were sectioned perpendicularly to the bonding surface using a water-cooled diamond disk. The sections were examined under a stereomicroscope (SZX12, OLYMPUS, Japan) connected to a digital camera Evolution MP 5.0 RTV (Color-Media Cybernetics, Canada) at magnification of 40x. The images of the sections were obtained and the length of silver particles deposited on the sealant-enamel interface was measured.  Figure 2 showed the schematic diagram of the study.

### 2.4. Statistical Analysis

The SBS and microleakage data were denoted as mean ± standard deviation. Differences among these groups were analyzed using ANOVA analysis. All statistical analyses were performed by using SPSS 22.0 (SPSS Inc., Chicago, IL, USA) and* p*<0.05 was considered as statistically significant.

## 3. Results

### 3.1. Bonding Interface

**Figure 3:**
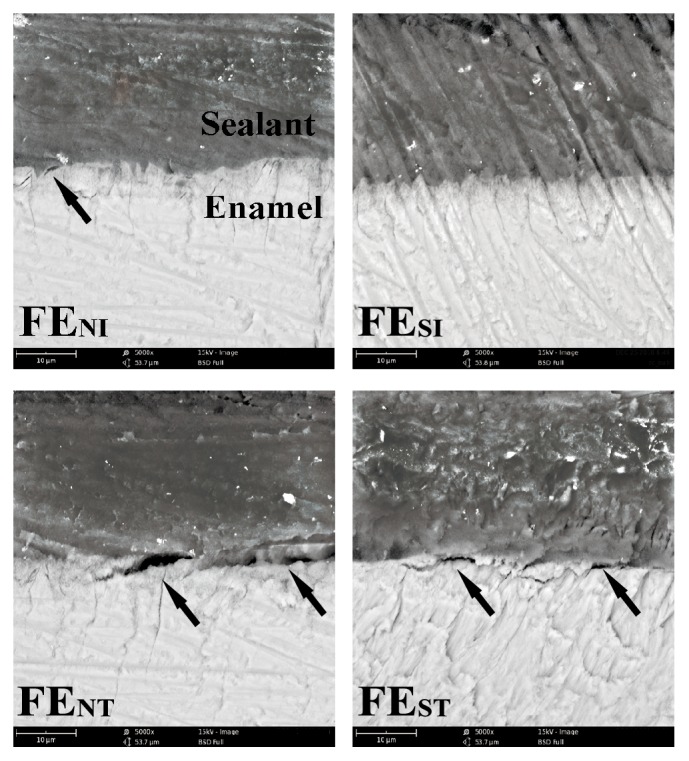
SEM images showed the bonding interface between sealant E and enamel at magnification of 5,000x.

Figure 3 showed the representative SEM images of the enamel-sealant interface of sealant E. No obvious voids in the interface were discovered in the subpressure groups before thermocycling (group FE_SI_), and meanwhile, the enamel and sealant fitted very well. By contrast, some voids (indicated by the arrow) were found in the interface of specimens in the nonsubpressure groups (groups FE_NI_ and FE_NT_) and subpressure groups after thermocycling (group FE_ST_).

**Figure 4:**
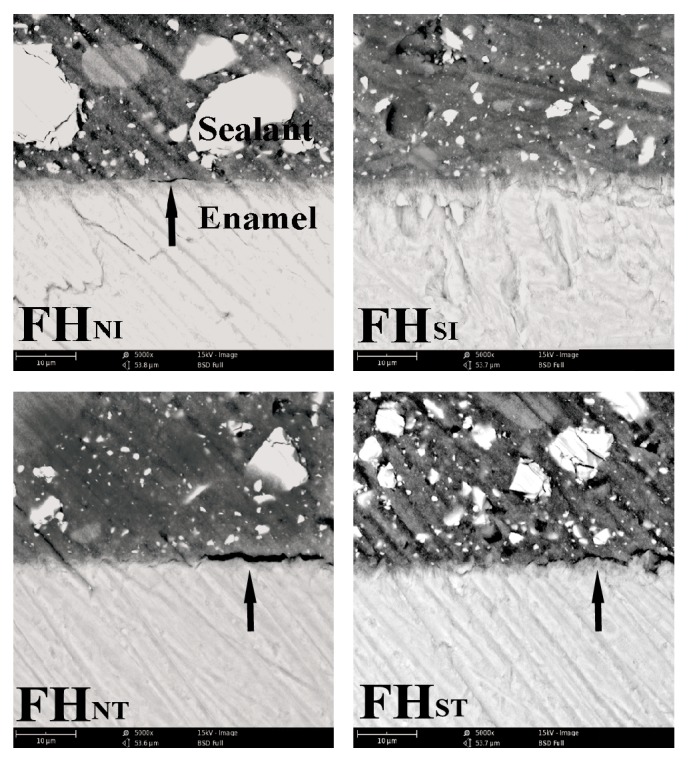
SEM images showed the bonding interface between sealant H and enamel at magnification of 5,000x.

Figure 4 revealed the representative SEM images of the enamel-sealant interface of sealant H. Similarly, there were not voids which were discovered obviously in the subpressure groups before thermocycling (group FH_SI_). By contrast, some voids (indicated by the arrow) were noticed in the interface of specimens in nonsubpressure groups (groups FH_NI_ and FH_NT_) and subpressure groups after thermocycling (group FH_ST_).

### 3.2. Shear Bond Strength

**Figure 5:**
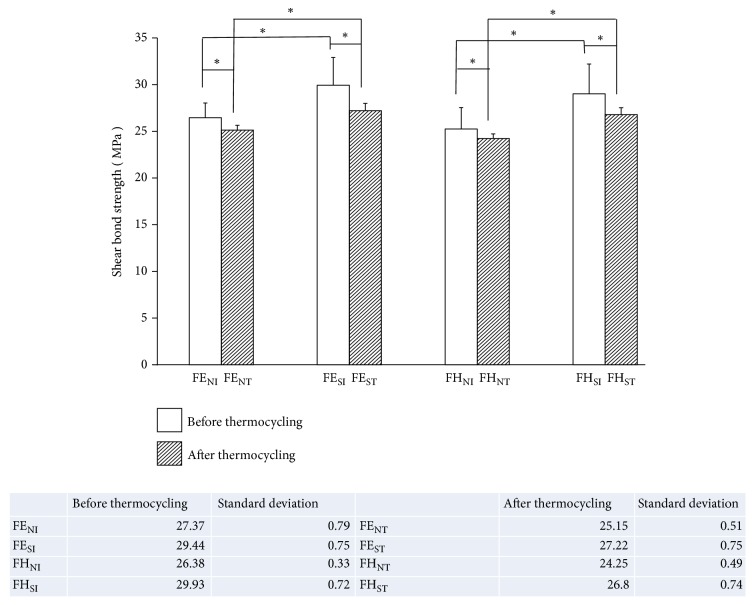
The SBS of specimens in each group.

Figure 5 showed the values of SBS of different groups in the form of mean ± standard deviations. In detail, the SBS was 26.47 ± 1.58 MPa for FE_NI_, 29.95 ± 2.96 MPa for FE_SI_, 25.27 ± 2.29 MPa for FH_NI_, 29.03 ± 3.19 MPa for FH_SI_, 25.15 ± 0.51 MPa for FE_NT_, 27.22 ± 0.77 MPa for FE_ST_, 24.25 ± 0.49 MPa for FH_NT_, and 26.80 ± 0.73 MPa for FH_ST_. The results clearly showed that samples in the subpressure groups had significantly higher SBS than specimens in the nonsubpressure groups regardless of thermocycling (*p*<0.05). In addition, specimens subjected to thermocycling had significantly decreased SBS compared to samples without aging (*p*<0.05). Meanwhile the SBS values in the nonsubpressure groups before thermocycling were still lower than those of the subpressure groups after thermocycling (*p*<0.05). Moreover, different sealant types had no significant effects on SBS of all samples (*p*>0.05).

### 3.3. Surface Fracture Types

Table 1 demonstrated the fracture types of each group after the SBS testing. Specimens in the subpressure groups had significantly higher proportions of mixed fractures and cohesive failure in the sealant than samples in the nonsubpressure groups (*p*<0.05). Moreover, the proportion of mixed fractures was the highest for samples in groups FE_NI_, FE_SI_, FH_NI_, FH_SI_, FE_NT_, FE_ST_, FH_NT_, and FH_ST_.

### 3.4. Microleakage Evaluation

**Figure 6:**
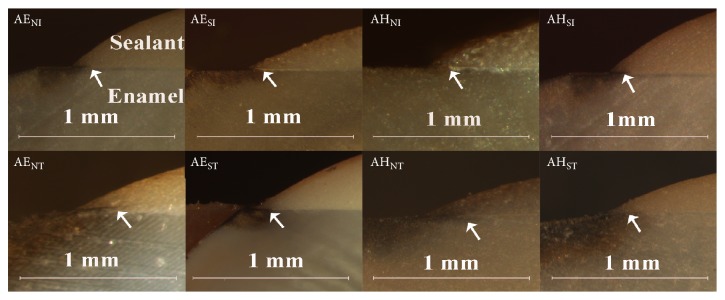
Images of microleakage of specimens with flat surfaces.

Figure 6 revealed the images of microleakage of samples in group A. Overall, specimens in the subpressure groups (AE_SI_ and AH_SI_) had significantly shallower silver depositions than samples in the nonsubpressure groups (AE_NI_ and AH_NI_) (*p*<0.05) and after thermocycling, these silver depositions infiltrated significantly deeper (*p*<0.05). In addition, sealant types had no significant effects on microleakage extent on the interface of enamel-sealant (*p*>0.05).

**Figure 7:**
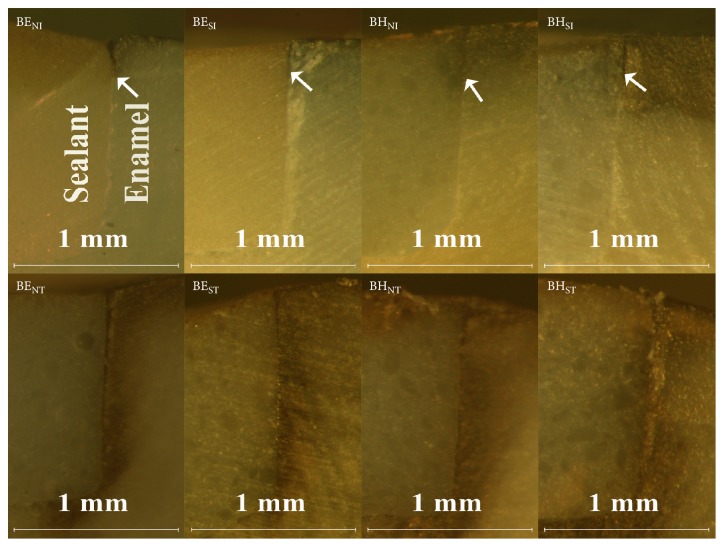
Images of microleakage of specimens with cavities.

Figure 7 showed the images of microleakage of samples in group B. Before thermocycling, samples in the subpressure groups (BE_SI_ and BH_SI_) had shallower microleakage than samples in the nonsubpressure groups (*p*<0.05). After thermocycling, silver depositions of samples in all groups appeared in the entire interface, which was significantly different from those of samples before thermocycling. Moreover, sealant types had no obvious effect on the microleakage (*p*>0.05).  Table 2 showed the length of microleakage of samples in groups A and B.

## 4. Discussion

Compared to no intervention, the effectiveness of sealants for controlling caries has been proven in clinical trials and summarized in systematic reviews. But it is still challenging to enhance the retention of the sealant and to reduce the microleakage [21]. Although numerous dental techniques have been explored to promote the bond strength of sealant-enamel, no established protocol has been found which can supply stable bonding at the present time [22, 23]. In addition, studies have proposed that voids could influence the bonding strength and microleakage [24]. This study evaluates the effects of subpressure on the SBS and microleakage of sealants before and after thermocycling. Based on the results of the study, the null hypotheses of this study are not rejected.

In this study, the vacuum chuck covers the enamel surface after the sealants are applied; the bubbles in the sealants and bonding interface are exhausted owing to the pressure gradient. When the subpressure is released, the sealants are pressed closer on the enamel. As a result, voids are not discovered in the bonding interface of samples in the subpressure groups. Furthermore, removal of voids leads to closer contact of sealant-enamel, increased bonding area, and enhanced mechanical locking and intermolecular forces. Therefore, SBS of the samples in the subpressure groups is superior to that of samples in the nonsubpressure groups.

Moreover, sealants are prone to fatigue aging because of temperature changes in the oral environment. In addition, the temperature in the oral environment varies from 5°C to 55°C in daily life. The thermocycling test (from 5°C to 55°C, 5000 cycles) is artificial simulation of the five-year effect of the sealant in the mouth to investigate the fatigue aging performance of sealants. The decreased SBS and increased microleakage depth after thermocycling are mainly due to the difference in the thermal expansion coefficients between the sealants and the enamel.

Notably, the subpressure application improved both the immediate enamel-sealant bonding and the bonding stability after thermocycling. The phenomenon is thanks to the superior performance of the subpressure in the sealant bonding. Although the SBS values of samples in the subpressure groups become lower after thermocycling, they are still higher than those of samples in the nonsubpressure groups before thermocycling. This phenomenon indicates that the subpressure technique is an effective method to improve the bonding against aging.

Voids are discovered in the subpressure group after thermocycling. This is possibly because of thermocycling-induced sealant shrinkage and interfacial cracks owing to difference in thermal expansion coefficients between the sealants and enamel.

For the sake of simulating the low and high C-factor pits and fissures, the flat enamel surface and round cavity are prepared to test the influences of subpressure and thermocycling on the microleakage of a pit-and-fissure sealant [25, 26]. Specimens of group A belong to the low C-factor to simulate the shallow pits and fissures. Before thermocycling, the microleakage extent of group A under the subpressure condition is shallower than samples under the nonsubpressure condition (*p* < 0.05). After thermocycling, the tendency is similar. Specimens of group B represent a high C-factor to simulate the deep pits and fissures. Before thermocycling, the microleakage extent of samples in group B under the nonsubpressure condition is deeper than that under the subpressure condition (*p* < 0.05).

However, the microleakage extent reaches the cavity bottom in specimens of group B after thermocycling, regardless of subpressure or nonsubpressure conditions (*p* > 0.05). These results indicate that subpressure can decrease the microleakage before and after thermocycling for the low C-factor specimens, while subpressure seems to only effectively decrease the microleakage before thermocycling for the high C-factor samples. The reason is considered as larger shrinkage is found in the interface of sealant-enamel after sealant curing for the high C-factor cavity [27, 28]. Particularly, the debonding pathway is susceptible to occurring in high C-factor specimens between the sealant and enamel after thermocycling. Therefore, the microleakage performances have no obvious difference for the specimens in group B regardless of subpressure or nonsubpressure conditions after thermocycling.

In this study, sealant types (sealant 3M ESPE Concise and sealant Helioseal F, codes E and H) have no significant effects on the bonding properties of sealants-enamel possibly by reason of similar curing shrinkage of the two sealants, although sealant F contains fillers. In the clinic, sealants usually contain little filler or are filler-free to keep fluidity [29]. But sealants with more fillers could resist curing shrinkage of sealants and resist wear. The subpressure can remove the voids in the bonding interface and keep the sealants contact closer to the enamel. Therefore, the higher amounts of fillers in sealants can be exploited in the future, which can be applied with the subpressure to resist curing shrinkage and wear. Further research is needed to test the effect of subpressure on higher viscosity pit-and-fissure sealants.

The subpressure condition is -0.1 MPa and 15-second duration in this study. Whether a stronger gradient and longer duration of subpressure could enhance bonding ability and resistance to microleakage of different sealants needs to be further explored.

## 5. Conclusions

The subpressure technique can effectively enhance the bonding of the sealant-enamel and reduce the microleakage on the interface of the sealant-enamel and has critical potential in the field of pit-and-fissure sealing against secondary caries and against sealant shedding.

## Figures and Tables

**Table 1:** Proportion (%) of different failure types of samples in each group after SBS test.

	Type of failure				
Group	IF	CE	CS	M	Sig.
FE_NI_	-	10	-	90	a f
FE_SI_	-	-	20	80	a b h
FE_NT_	10	-	-	90	i
FE_ST_	-	10	-	90	b j
FH_NI_	-	-	-	100	c d f
FH_SI_	-	-	10	90	c e g h
FH_NT_	10	10	-	80	d f i
FH_ST_	-	-	20	80	e g j

Abbreviations: IF, interface failure; CE, cohesive failure in the enamel; CS, cohesive failure in the sealant; M, mixed failure. Identical letters indicate their difference is significant (*p*<0.05).

**Table 2:** Depth of microleakage of specimens in each group.

Group	Microleakage depth (mean ± SD, mm)	
	Before thermocycling	After thermocycling
Group AE_N_	0.24±0.02^a^	0.30±0.03^c^
Group AE_S_	0.15±0.04^b^	0.22±0.02^d^
Group AH_N_	0.23±0.01^a^	0.31±0.02^c^
Group AH_S_	0.15±0.01^b^	0.23±0.02^d^

Group BE_N_	0.23±0.02^e^	+^g^
Group BE_S_	0.16±0.03^f^	+^g^
Group BH_N_	0.23±0.03^e^	+^g^
Group BH_S_	0.15±0.01^f^	+^g^

Identical letters (a, b, c, d, e, f, and g) indicated their difference was not significant (*p* > 0.05). + indicated the silver depositions appeared in the entire interface.

## Data Availability

The data used to support the findings of this study are available from the corresponding author upon request.
